# Genetically regulated expression in late-onset Alzheimer’s disease implicates risk genes within known and novel loci

**DOI:** 10.1038/s41398-021-01677-0

**Published:** 2021-12-06

**Authors:** Hung-Hsin Chen, Lauren E. Petty, Jin Sha, Yi Zhao, Amanda Kuzma, Otto Valladares, Alzheimer’s Disease Genetics Consortium, International Genomics of Alzheimer’s Project, William Bush, Adam C. Naj, Eric R. Gamazon, Jennifer E. Below

**Affiliations:** 1 Vanderbilt Genetics Institute and Division of Genetic Medicine, Department of Medicine, Vanderbilt University Medical Center, Nashville, TN USA; 2 Department of Biostatistics, Epidemiology, and Informatics, Perelman School of Medicine, University of Pennsylvania, Philadelphia, PA USA; 3 Department of Pathology and Laboratory Medicine, Perelman School of Medicine, University of Pennsylvania, Philadelphia, PA USA; 4 Department of Population & Quantitative Health Sciences, School of Medicine, Case Western Reserve University, Cleveland, OH USA

**Keywords:** Genomics, Molecular neuroscience

## Abstract

Late-onset Alzheimer disease (LOAD) is highly polygenic, with a heritability estimated between 40 and 80%, yet risk variants identified in genome-wide studies explain only ~8% of phenotypic variance. Due to its increased power and interpretability, genetically regulated expression (GReX) analysis is an emerging approach to investigate the genetic mechanisms of complex diseases. Here, we conducted GReX analysis within and across 51 tissues on 39 LOAD GWAS data sets comprising 58,713 cases and controls from the Alzheimer’s Disease Genetics Consortium (ADGC) and the International Genomics of Alzheimer’s Project (IGAP). Meta-analysis across studies identified 216 unique significant genes, including 72 with no previously reported LOAD GWAS associations. Cross-brain-tissue and cross-GTEx models revealed eight additional genes significantly associated with LOAD. Conditional analysis of previously reported loci using established LOAD-risk variants identified eight genes reaching genome-wide significance independent of known signals. Moreover, the proportion of SNP-based heritability is highly enriched in genes identified by GReX analysis. In summary, GReX-based meta-analysis in LOAD identifies 216 genes (including 72 novel genes), illuminating the role of gene regulatory models in LOAD.

## Introduction

Late-onset Alzheimer’s disease (LOAD) is the most common neurodegenerative disease in the world, occurring in >35% of individuals age 85 years and older [1], and it is the sixth most common cause of death in the US. LOAD has a substantial genetic component, with heritability estimated to be between 40 and 80% [2–4]. Despite this high heritability and numerous genome-wide studies conducted to date, known LOAD-associated single nucleotide polymorphisms (SNPs) explain only 30.62% of the genetic variance [5], with the majority of risk attributed to variants within *APOE* [5, 6]. Much of the SNP-based heritability of LOAD remains unmapped to causal genetic factors; this so-called “missing heritability”, is common to many complex diseases [7]. Recently developed methods leveraging transcriptomic data have powered the identification of novel genetic risk factors, increasing the proportion of heritability explained by known loci and expanding our understanding of the biological processes underlying disease, such as cardiometabolic diseases and neuropsychiatric traits [8–10].

In the most recent large-scale meta-analysis of LOAD GWAS a total of 25 genes were identified, including five novel genes, *IQCK*, *ACE*, *ADAM10*, *ADAMTS1*, and *WWOX* [11]. Another recent meta-analysis, maximized the sample size by including potential AD cases based on parental AD status, identifying 13 novel loci [12]. Although these genes have been associated with LOAD by the position of GWAS-identified SNPs, understanding and interpreting the biological implications of LOAD associations is a difficult challenge due to our limited knowledge of the underlying mechanisms. Among the 1073 GWAS-identified LOAD variants, only 2% are located in gene coding regions, and most do not have a clear molecular function for LOAD [11]. Gene expression studies provide an opportunity to investigate an important class of molecular mediators and their consequent effects on disease outcomes. Gene expression is regulated by complex mechanisms involving various factors, e.g., genetic variation, age, and environmental stimuli. The importance of gene expression is demonstrated by the enrichment of GWAS-identified loci in expression quantitative trait loci (eQTLs) [13]. As previously estimated, over 70% of LOAD variant have the potential to regulate the gene expression across all tissues [11]. A previous transcriptome study in human brain tissues has identified 207 differentially expressed genes [14, 15]. In addition to identifying more novel LOAD genes, transcriptome-informed studies offer a potential genetic mechanism of disease. Previously, rs1057233 was reported as a *CELF1* LOAD locus [16], but a further gene expression study suggests it may cause LOAD via affecting SPI1 expression instead of CELF1 [17]. Therefore, investigating the association between gene expression and traits of interest can be an effective way to understand the functional mechanism of GWAS loci.

Although studying gene expression has improved our knowledge about the genetic mechanism of LOAD, the difficulty of collecting LOAD relevant tissues historically has limited the sample sizes available for analysis, and the complexity of gene expression regulation is also heavily affected by environment and other uncontrollable factors. Genetically regulated expression (GReX) association is an emerging method to test the association of gene expression, imputed using common genetic variants, with phenotype. Since genetic influence on phenotype is lifelong, GReX reduces noise from temporal and environmental factors, and instead identifies the direct impact of genetic effects that influence gene expression on trait [18]. Also, the imputed GReX is based on genetic variants, so we can leverage the sample size of previous genetic LOAD studies to increase the statistical power of this gene-based analysis. Moreover, GReX aggregates genetic variants into a gene-level functional unit, based on gene expression. As a result, GReX results are intrinsically related to function in contrast to single variant GWAS where the results are often found in intergenic regions and the relevant gene remains unclear. The gene-level test also has the effect of increasing the statistical power by reducing multiple testing burden.

In this study, we imputed tissue-specific GReX using Predixcan for 12,162 LOAD cases and 13,614 healthy controls from 31 epidemiological studies of LOAD. PrediXcan trains models of gene expression in a transcriptome reference panel linked to whole-genome data, such as the Genotype Tissue Expression Project (GTEx) and leverages the models to impute GReX in independent genomic data [18]. In addition, S-PrediXcan was applied for another eight LOAD studies (8451 cases and 24,044 controls) for which individual genotype data were not available. S-PrediXcan was developed to use GWAS summary statistics instead, and estimates the gene-trait association based on these statistics and reference linkage disequilibrium structure [19]. With both SNP-based and GWAS summary statistic-based approaches, data on a total of 58,713 individuals were examined in this study. Although summary-based GReX has been successfully applied in various traits [20–22], the fact that the allele frequency and linkage disequilibrium data come from an external population may reduce power to detect gene-trait associations, and its effects have not been determined. Therefore, we compared the sensitivity of genotype-based and summary statistic-based GReX methods to quantify the benefit of using an internal LD structure. In addition to tissue-specific GReX analysis, cross-tissue GReX has been proposed to increase the power to detect gene-trait associations [23]. Due to the high correlation of gene expression across tissues [24], cross-tissue GReX models extract the co-expression across selected tissues, thereby allowing for the identification of more genes by aggregating their effects, especially for the identification of genes with modest signals in several tissues that do not meet single-tissue significance thresholds. Therefore, we also implemented two cross-tissue models, one including all brain tissues and one including all available tissues. Finally, to quantify the improvement of GReX approaches over traditional GWAS, we estimated the proportion of heritability captured by our identified LOAD genes, demonstrating that the major component of LOAD SNP-based heritability is captured by this approach.

## Subjects and methods

### ADGC Subjects

The ADGC GWAS data comprised 31 studies with different sample sizes and ratios of controls to cases (Supplementary Table 1). Seven waves of subjects were selected from the National Institute on Aging (NIA) Alzheimer’s Disease Centers (ADCs), including the Adult Changes in Thought (ACT) study [25], the Alzheimer Disease Neuroimaging Initiative (ADNI) study [26], the National Institute on Aging Late-Onset Alzheimer’s Disease Family (NIA-LOAD) study [27], the Mayo Clinic Jacksonville (MAYO), the Multi Institutional Research of Alzheimer Genetic Epidemiology (MIRAGE) Study [28], Oregon Health and Science University (OHSU), the Rush University Religious Orders Study/Memory and Aging Project (ROS/MAP) [29], the Translational Genomics Research Institute series 2 (TGEN2) [30], the University of Miami/Case Western Reserve University/Mt. Sinai School of Medicine (UM/CWRU/MSSM), University of Pittsburgh (UPitt) [31], two waves from Washington University (WASHU), the Multi-Site Collaborative Study for Genotype-Phenotype Associations in Alzheimer’s disease (GenADA) [28], the Universitätsklinikum des Saarlandes (UKS), and the Netherlands Brain Bank (NBB) [32], Biomarkers of Cognitive Decline Among Normal Individuals: the BIOCARD cohort (BIOCARD), Chicago Health and Aging Project (CHAP2), Einstein Aging Study (EAS), Mayo Clinic (RMAYO), Washington Heights-Inwood Community Aging Project (WHICAP). A detailed description of inclusion and exclusion criteria has been provided in previous studies [11, 32, 33].

Genotyping was done with either Illumina or Affymetrix high-density SNP microarrays. The criteria for minimal call rate and minor allele frequency (MAF) were 0.95 and 0.02 for the Illumina chip, and 0.98 and 0.01 for Affymetrix. Genotype imputation was conducted using the HRC r1.1 as the reference panel and minimac3 for imputation on the University of Michigan Imputation Server [34]. Variants with MAF > 0.05 and imputation score (*R*^2^ for MaCH/Minimac3 > 0.5) were included in further analyses. A total of 25,776 samples of European ancestry, comprising 12,162 cases and 13,614 controls, were carried forward in the ADGC analysis [16].

### Imputing tissue-specific expression using PrediXcan models

Tissue-specific GReX in ADGC was imputed using PrediXcan with publicly-available gene expression imputation models built-in reference transcriptome data sets [18]. In total, 51 tissue-specific models were used, including the whole blood model for DGN [35], the dorsolateral prefrontal cortex (DLPFC) model from the CommonMind Consortium [21, 36], and another 49 tissues models from the Genotype-Tissue Expression (GTEx) project (version v8) [37, 38]. DGN and DLPFC models were trained using an elastic net approach, and the other models from GTEx v8 used multivariate adaptive shrinkage (MASHR) [38]. These PrediXcan models leveraged cross-tissue information in the model building step by applying a Bayesian methodology, the multivariate adaptive shrinkage model (MASHR), which allows for sparse effects and correlation in effect sizes across tissues [38].

### Firth regression

Maximum likelihood estimation from conventional logistic regression may suffer from bias due to the presence of rare events, such as in extremely unbalanced data sets. For this reason, we used Firth logistic regression for GReX association testing, which is less sensitive to bias due to case–control imbalance [39]. To test the association between imputed gene expression level and disease status, logistic Firth regression was performed within each study using R package, logistf, with covariate adjustment for sex, age (age-at-onset for cases and age-at-last-exam or age-at-death for controls), and principal components analysis (PCA) to correct for population structure [40] (from 2 to 4 components for each dataset, based on total variance explained, as described in Naj et al. [33]).

### Conditional analyses in known LOAD-associated regions

Because our PrediXcan results validated many previously reported LOAD regions, for all PrediXcan significant genes located 10 Mb upstream or downstream of a known LOAD-risk SNP reported in Naj et al. [41], we conducted conditional analyses two ways. Firstly, we adjusted for the nearby previously reported SNP by including the additive genotype for each SNP as a covariate in the Firth regression model. Secondly, we similarly adjusted for GReX of the gene reported for the known SNP to characterize independent residual effects. The known risk SNPs included 29 total SNPs located in 28 genes (Supplementary Table 2) [41]. If the previously known SNP was not available in the set of high-quality imputed variants in ADGC, a tag SNP was chosen, prioritizing SNPs in strongest linkage disequilibrium with the risk SNP (*r*^2^ > 0.6 based on the 1000 Genomes Project CEU reference population). No SNPs with *r*^2^ > 0.6 were available in most studies for eight SNPs of interest, and thus we did not perform SNP-adjusted conditional analysis for rs75932628 (*TREM2*), rs11218343 (*SORL1*), rs74615166 (*TRIP4*), rs138190086 (*ACE*), rs8093731 (*DSG2*), rs145999145 (*PLD3*), rs63750847 (*APP*), and rs7412 (*APOE*) (though data was available for the other *APOE* ε2/ε3/ε4 haplotype SNP, rs429358). Conditional analyses were performed using logistic Firth regression in R, adjusting for the same covariates used in the primary analysis, as well as the dosage of the LOAD-risk SNP or the tissue-specific GReX of the gene reported for the known SNP.

### Cross-tissue analyses

To evaluate genetic effects across tissues, we used multivariate logistic Firth regression. We considered two different cross-tissue analyses: (1) cross-all tissues, combining expression data from all available GTEx tissues (Supplementary Table 3), and (2) cross-brain tissues, combining expression data from all available brain tissues in GTEx (Supplementary Table 3). Only genes with prediction models in at least five (cross-all) or three (cross-brain) tissues were included in these analyses. Because we expect correlation in expression across tissues, to avoid collinearity, we computed principal components for each gene using all available predicted gene expression levels across (1) all tissues and (2) brain tissues only. Principal components sufficient to explain >80% of variance were carried forward in association tests. We conducted Firth regression including covariate adjustment for sex, age-at-onset/age-at-last-exam, SNP-based principal components (i.e., genetic ancestry) for the reduced model, **M**_**reduced**_, and, in addition, the expression-based principal components for the full model, **M**_**full**_. Significance was tested by comparing **M**_**full**_ and **M**_**reduced**_ using a likelihood ratio test:

$$L = 2({\mathrm{ln}}(L_{{\mathrm{full}}}) - {\mathrm{ln}}(L_{{\mathrm{reduced}}}))$$

Here $L_{{\mathrm{full}}}$ and $L_{{\mathrm{reduced}}}$ are the likelihood for the full and reduced models, respectively. This likelihood ratio statistic has an asymptotic *χ*^2^ distribution under the null hypothesis. We calculated the *p*-value using a *χ*^2^ test in which the number of degrees of freedom is equal to the number of principal components, which varies by gene.

### IGAP subjects

The International Genomics of Alzheimer’s Project (IGAP) data used here comprised eight European GWAS from seven studies with LOAD data: four prospective cohort studies from the Cohorts for Heart and Aging Research in Genomic Epidemiology (CHARGE) Consortium [42], including the Atherosclerosis Risk in Communities (ARIC) Study [43], Cardiovascular Health Study (CHS) [44], the Framingham Heart Study (FHS) [45], and two waves of subjects from the Rotterdam Study (RS) [46]; the Genetic and Environmental Risk in Alzheimer’s Disease (GERAD) Consortium [47]; the European Alzheimer’s Disease Initiative (EADI) [48], and the Bonn Study [49, 50]. A previous meta-analysis of these and other LOAD GWAS has described sampling and phenotyping procedures as well as demographic characteristics for each of these studies [11, 16]. Genotyping and association analysis methods are also detailed in Kunkle et al. [11]. Summary statistics from each study were cleaned to remove low frequency and low imputation quality using the same inclusion thresholds used for ADGC (MAF < 0.01 and *R*^2^ < 0.5).

### S-PrediXcan and S-MultiXcan

S-PrediXcan [19] infers GReX-trait association from GWAS summary statistics using the same expression prediction models as for PrediXcan, as well as linkage disequilibrium data from a reference population (here 1000 Genomes Project), which captures covariance of variants in the prediction model. Summary statistics from the IGAP GWAS were pruned to remove variants with low frequency (MAF < 0.01) and low imputation quality (*R*^2^ < 0.5) and supplied as input to S-PrediXcan. We inferred cross-tissue GReX-trait association based on summary statistics in IGAP using S-MultiXcan. S-MultiXcan extracts cross-tissue GReX principal components, and evaluates statistical significance by assessing model fitness using an *F*-test. The same criteria for allele frequency and imputation quality were applied. Two cross-tissue models were implemented, cross-all GTEx tissues and cross-GTEx-brain tissues.

### Meta-analysis and multiple testing correction

We performed fixed-effects meta-analysis of the single-tissue and the cross-tissue results, separately, across studies (Supplementary Table 1). Let $z_i$ and $N_i$ be the *z*-score for a gene and sample size respectively from study *i*. The weighted *z*-score statistic $Z_{{\mathrm{combined}}}$ is defined as:

$$Z_{{\mathrm{combined}}} = {\sum} {(N_iz_i)/\sqrt {\Sigma N_i^2} }$$

This statistic is distributed as a standard normal distribution, $N(0,1)$, under the null hypothesis, yielding a *p*-value. We used METAL [51] for the single-tissue results and the *metap* R package [52] implementation for the cross-tissue model.

We applied the Benjamini–Hochberg method for study-wide multiple testing correction [53]. The total number of tests for discovery was 733,844 (from single-tissue and cross-tissue analyses). We used B–H adjusted *p*-value < 0.05 as the significance cutoff, corresponding to an original study-wide *p*-value significance threshold of *p* = 1.02 × 10^-4^.

### Causal inference

We conducted the FOCUS to clarify the importance of genes within the same linkage disequilibrium region [54]. We tested all the multivariate adaptive shrinkage models from GTEx v8, and the linkage disequilibrium region was determined by the European ancestry population in 1000 Genomes. Only the regions containing GWAS *p*-value <1 × 10^−5^ were used for the fine mapping. The LOAD GWAS was produced with all ADGC studies. We used the marginal posterior inclusion probability (PIP) to determine the gene’s importance, and used Spearman’s rank correlation test to compare it with the *Z*-score from our GReX association test in ADGC. Furthermore, we used Mendelian randomization to distinguish the SNPs’ effect on LOAD whether through gene expression or not. The same ADGC’s GWAS was used, and the eQTLs associations were from GTEx v.8. We applied the median-based Mendelian randomization (mr_median) in the R package, “MendelianRandomization”, on our identified LOAD genes [55, 56].

### Sensitivity analyses

To facilitate a direct comparison of the PrediXcan and S-PrediXcan approaches, we completed an S-PrediXcan analysis using the same ADGC data/samples used for the PrediXcan analysis. Summary statistics from the ADGC GWAS performed above were pruned to remove variants with low frequency (MAF < 0.01) and low imputation quality (*R*^2^ < 0.5) and supplied as input to S-PrediXcan.

To evaluate the performance of individual tissue-specific models, we used the true positive rate of the association test from GReX analysis in each tissue, using the method described in Storey et al. [57].

### Heritability analyses

We used linkage disequilibrium score regression (as implemented in LDSC [58]) to estimate the heritability of LOAD and the proportion of heritability attributable to our identified LOAD genes. LDSC estimates heritability based on the relationship between GWAS summary statistics and linkage disequilibrium. To quantify the overall increase in heritability explained in our study, we used LDSC in summary statistics from a previous LOAD GWAS [16]. We calculated the proportion of heritability explained by all genes identified in our study, conducting an LDSC analysis including only SNPs within 1 Mb of our identified genes, and compared this to the previous estimate of heritability. We also estimated heritability explained by the *APOE* region alone, including only SNPs within 10 Mb of *APOE*, and compared this to the estimated heritability outside of the *APOE* region, including all SNPs within 1 Mb of our identified genes but not within 10 Mb of *APOE*. Finally, we calculated heritability from different tissue-specific findings, including SNPs within 1 Mb of our findings from (1) only whole blood and (2) only brain tissues.

## Results

We included two sources of data in this study. First, we utilized imputed genotype data from Alzheimer’s Disease Genetics Consortium (ADGC). ADGC contains 31 LOAD epidemiological studies with 12,162 LOAD cases and 13,614 controls (Supplementary Table 1). Genome-wide GReX were imputed by PrediXcan with the whole blood model from Depression Susceptibility Genes and Networks (DGN), dorsolateral prefrontal cortex model from CommonMind Consortium (DLPFC), and 49 tissue-specific models from GTEx v8. Second, we also analyzed GWAS summary results from the International Genomics of Alzheimer’s Project (IGAP) after excluding samples overlapping with ADGC, comprising another eight LOAD studies with 8451 cases and 24,044 controls. We applied S-PrediXcan to these summary statistics to identify gene-level associations with LOAD. Across both data sets, a total of 23,625 unique genes across 51 tissue-specific models were tested. To determine study-wide significance, we used the Benjamini–Hochberg procedure to control the false discovery rate. Genes with an adjusted *p*-value of <0.05 were considered significant (*p*-value < 1.02 × 10^-4^ across 733,844 tests).

**Fig. 1:**
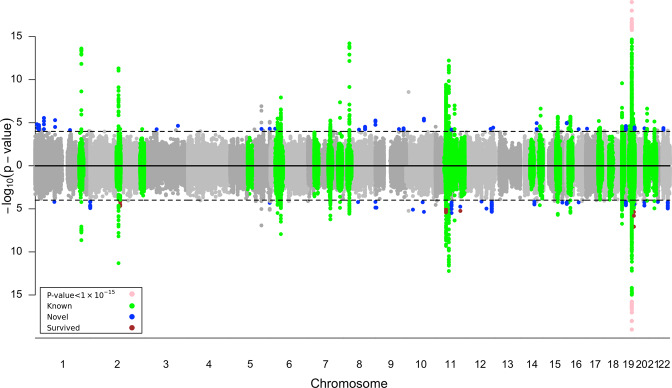
Miami plot of tissue-specific GReX association tests with LOAD from meta-analysis of AGDC and IGAP (top) or ADGC-only (bottom). The green region labels the ±10 Mb of known AD loci, the blue dot indicates the novel gene, which locates outside the known AD region (green) and is never reported in previous GWAS, and the brown dot in ADGC-only meta-analysis suggests the gene holds significance in conditional analysis. *P*-values less than 1 × 10^−15^ were truncated (pink dot) to improve the clarity of peaks in other, less significant regions.

In the 51 single-tissue models, we identified 1459 tissue-specific GReX from 208 unique genes significantly associated with LOAD (Fig. 1, Table 1, and Supplementary Table 4). The strongest gene association identified, *TOMM40*, falls within a large region of 43 significantly associated genes encompassing *APOE* and spanning 10 Mb in either direction (lead signals for *TOMM40* were observed in GTEx V8 skin tissues, *p*-value = 1.28 × 10^−600^). After adjusting for known LOAD SNPs nearby, 11 unique genes maintained their significance, including *LILRA5* from the *APOE* region, *TMEM163* from the *BIN1* region, *SIK2* from the *SORL1* region, and another 8 genes from the *CELF1* region (Table 2). *TOMM40* was not statistically significant after conditional analysis. In addition, 72 genes are novel LOAD genes that are neither reported in previous GWAS [59] (Table 1) nor within 10 Mb of 31 well-known LOAD SNPs from Naj et al. [41] (Supplementary Table 2).

**Table 1:** Strongest tissue-specific associations of novel LOAD genes.

		ADGC & IGAP			ADGC			IGAP		
Genes	Tissue	*N*	*Z*	*p*-value	*N*	*Z*	*p*-value	*N*	*Z*	*p*-value
*TRIT1*	Brain amygdala	58,713	4.615	3.92E-06	25,776	3.025	2.49E-03	32,937	3.487	4.89E-04
*TSPAN14*	Cells cultured fibroblasts	58,624	4.572	4.83E-06	25,687	2.224	2.61E-02	32,937	4.136	3.54E-05
*LRRC8D*	Testis	58,713	−4.493	7.02E-06	25,776	−3.599	3.19E-04	32,937	−2.815	4.88E-03
*SHARPIN*	Colon transverse	58,713	4.472	7.76E-06	25,776	3.426	6.13E-04	32,937	2.94	3.29E-03
*MYCL*	Artery coronary	58,713	−4.437	9.13E-06	25,776	−2.947	3.21E-03	32,937	−3.316	9.12E-04
*CLUAP1*	Cells cultured fibroblasts	56,140	4.333	1.47E-05	24,133	3.948	7.89E-05	32,007	2.31	2.09E-02
*LTBP2*	Prostate	58,713	−4.314	1.60E-05	25,776	−2.677	7.44E-03	32,937	−3.392	6.94E-04
*RERE*	Heart atrial appendage	58,713	−4.249	2.15E-05	25,776	−1.44	1.50E-01	32,937	−4.399	1.09E-05
*ARHGEF10L*	Artery tibial	58,684	−4.198	2.69E-05	25,747	−2.698	6.97E-03	32,937	−3.218	1.29E-03
*TMEM14EP*	DGN-WB	58,713	4.155	3.25E-05	25,776	3.344	8.27E-04	32,937	2.59	9.60E-03
*SSBP4*	Esophagus muscularis	58,713	−4.142	3.44E-05	25,776	−3.937	8.24E-05	32,937	−2.048	4.06E-02
*MTDH*	Adipose subcutaneous	58,449	4.094	4.24E-05	25,512	3.338	8.42E-04	32,937	2.515	1.19E-02
*LRRC25*	Artery tibial	58,713	−4.081	4.48E-05	25,776	−2.8	5.11E-03	32,937	−2.972	2.96E-03
*TRIB3*	Breast mammary tissue	58,713	4.059	4.94E-05	25,776	3.234	1.22E-03	32,937	2.558	1.05E-02
*AC007228.9*	Muscle skeletal	56,813	−4.056	5.00E-05	23,876	−2.445	1.45E-02	32,937	−3.245	1.17E-03
*SCARB1*	Muscle skeletal	58,713	4.041	5.33E-05	25,776	2.576	1.00E-02	32,937	3.116	1.83E-03
*EXOSC10*	Skin not sun-exposed suprapubic	58,713	4.027	5.64E-05	25,776	1.648	9.95E-02	32,937	3.92	8.86E-05
*YDJC*	Brain putamen basal ganglia	58,713	−4.01	6.08E-05	25,776	−3.581	3.43E-04	32,937	−2.186	2.88E-02
*VPS13D*	Brain cortex	58,713	−4.004	6.22E-05	25,776	−1.297	1.95E-01	32,937	−4.199	2.68E-05
*ADA*	DLPFC	58,713	3.994	6.51E-05	25,776	2.25	2.45E-02	32,937	3.342	8.32E-04
*AP000553.3*	Nerve tibial	58,713	3.993	6.52E-05	25,776	3.487	4.89E-04	32,937	2.247	2.46E-02
*CCDC116*	Brain spinal cord cervical c-1	58,713	3.993	6.52E-05	25,776	3.487	4.89E-04	32,937	2.247	2.46E-02
*MVB12B*	Artery aorta	58,713	−3.993	6.52E-05	25,776	−2.155	3.12E-02	32,937	−3.425	6.14E-04
*NBAT1*	Artery aorta	58,713	−3.973	7.09E-05	25,776	−2.623	8.71E-03	32,937	−2.984	2.84E-03
*RABEP1*	Adipose subcutaneous	58,713	3.973	7.10E-05	25,776	2.779	5.46E-03	32,937	2.846	4.42E-03
*DDX41*	Brain putamen basal ganglia	52,443	3.972	7.12E-05	23,391	3.442	5.77E-04	29,052	2.249	2.45E-02
*SDS*	Brain frontal cortex BA9	58,713	−3.959	7.54E-05	25,776	−2.147	3.18E-02	32,937	−3.386	7.10E-04
*UBE2L3*	DGN-WB	58,713	−3.956	7.61E-05	25,776	−3.584	3.38E-04	32,937	−2.112	3.47E-02
*HARS1*	DGN-WB	58,713	−3.955	7.64E-05	25,776	−2.201	2.77E-02	32,937	−3.334	8.57E-04
*AREL1*	Brain cerebellar hemisphere	58,713	3.948	7.90E-05	25,776	3.037	2.39E-03	32,937	2.584	9.77E-03
*MFSD2A*	Minor salivary gland	58,713	3.946	7.96E-05	25,776	2.959	3.09E-03	32,937	2.65	8.04E-03
*FKTN*	Brain caudate basal ganglia	58,713	3.944	8.02E-05	25,776	1.11	2.67E-01	32,937	4.284	1.84E-05
*MT1G*	Brain frontal cortex BA9	58,713	3.941	8.13E-05	25,776	0.741	4.58E-01	32,937	4.605	4.12E-06
*ARHGEF3*	DLPFC	58,713	3.934	8.34E-05	25,776	3.067	2.16E-03	32,937	2.54	1.11E-02
*FOLR2*	Minor salivary gland	58,351	3.918	8.92E-05	25,414	4.577	4.71E-06	32,937	1.195	2.32E-01
*TRAM1*	Esophagus gastroesophageal junction	58,713	−3.912	9.14E-05	25,776	−3.57	3.57E-04	32,937	−2.066	3.89E-02
*NTRK1*	Artery tibial	58,713	3.886	1.02E-04	25,776	2.197	2.80E-02	32,937	3.245	1.17E-03
*CLUL1*	Brain caudate basal ganglia	58,713	−3.827	1.30E-04	25,776	−1.158	2.47E-01	32,937	−4.085	4.42E-05
*ACP1*	Testis	58,713	−3.802	1.43E-04	25,776	−3.889	1.01E-04	32,937	−1.636	1.02E-01
*L3MBTL2*	Heart atrial appendage	58,713	3.783	1.55E-04	25,776	4.126	3.69E-05	32,937	1.4	1.61E-01
*CHADL*	Heart left ventricle	58,713	−3.773	1.62E-04	25,776	−4.201	2.66E-05	32,937	−1.321	1.87E-01
*OSER1*	DLPFC	58,713	−3.753	1.75E-04	25,776	−3.947	7.92E-05	32,937	−1.52	1.29E-01
*EP300*	Lung	58,713	3.715	2.03E-04	25,776	4.318	1.58E-05	32,937	1.141	2.54E-01
*INPPL1*	Ovary	58,351	3.686	2.28E-04	25,414	4.354	1.34E-05	32,937	1.081	2.80E-01
*PATJ*	Esophagus mucosa	58,713	3.683	2.30E-04	25,776	1.138	2.55E-01	32,937	3.911	9.21E-05
*RILPL2*	Lung	57,358	−3.621	2.94E-04	24,421	−0.259	7.95E-01	32,937	−4.555	5.25E-06
*SH3YL1*	Adrenal gland	58,713	3.616	2.99E-04	25,776	3.909	9.27E-05	32,937	1.37	1.71E-01
*COLCA2*	Stomach	58,713	3.616	3.00E-04	25,776	4.206	2.60E-05	32,937	1.106	2.69E-01
*ALKAL2*	Brain substantia nigra	58,713	3.583	3.40E-04	25,776	3.898	9.69E-05	32,937	1.335	1.82E-01
*GDAP1L1*	Artery tibial	58,713	3.575	3.51E-04	25,776	4.141	3.46E-05	32,937	1.11	2.67E-01
*ECE1*	Heart left ventricle	58,713	−3.574	3.52E-04	25,776	−0.686	4.93E-01	32,937	−4.164	3.12E-05
*NAV3*	Esophagus gastroesophageal junction	58,713	−3.567	3.61E-04	25,776	−0.991	3.22E-01	32,937	−3.887	1.02E-04
*NPC2*	Brain hippocampus	58,713	−3.467	5.26E-04	25,776	−0.133	8.94E-01	32,937	−4.511	6.44E-06
*MAP3K7*	Pancreas	58,713	−3.43	6.04E-04	25,776	−0.721	4.71E-01	32,937	−3.941	8.11E-05
*RBCK1*	Testis	58,713	3.389	7.02E-04	25,776	0.108	9.14E-01	32,937	4.429	9.46E-06
*PHB2*	Brain cortex	58,713	−3.171	1.52E-03	25,776	−0.378	7.06E-01	32,937	−3.9	9.62E-05
*EMG1*	Skin sun-exposed lower leg	58,713	3.138	1.70E-03	25,776	0.183	8.55E-01	32,937	4.029	5.61E-05
*AC026367.3*	Cells cultured fibroblasts	58,713	−3.099	1.94E-03	25,776	−4.513	6.40E-06	32,937	−0.146	8.84E-01
*TBC1D1*	Cells cultured fibroblasts	58,713	3.057	2.23E-03	25,776	0.161	8.72E-01	32,937	3.94	8.16E-05
*RABEPK*	Brain substantia nigra	35,698	3.013	2.58E-03	19,912	0.428	6.69E-01	15,786	4.051	5.10E-05
*AC026367.2*	Brain frontal cortex BA9	58,713	−2.985	2.84E-03	25,776	−4.364	1.28E-05	32,937	−0.124	9.01E-01
*ZNF295−AS1*	Minor salivary gland	58,713	−2.882	3.96E-03	25,776	−3.91	9.23E-05	32,937	−0.389	6.98E-01
*KAT6B*	Cells cultured fibroblasts	58,624	2.842	4.49E-03	25,687	3.928	8.58E-05	32,937	0.323	7.47E-01
*SPATA6L*	Brain cortex	58,713	2.832	4.63E-03	25,776	4.27	1.95E-05	32,937	0.003	9.98E-01
*SUDS3*	Esophagus mucosa	58,713	2.733	6.28E-03	25,776	4.169	3.07E-05	32,937	−0.039	9.69E-01
*TMCC3*	Brain cortex	58,713	−2.687	7.22E-03	25,776	−4.329	1.50E-05	32,937	0.243	8.08E-01
*HSPA2*	Colon sigmoid	58,175	2.521	1.17E-02	25,238	4.071	4.68E-05	32,937	−0.213	8.31E-01
*RRS1*	Heart left ventricle	58,713	−2.152	3.14E-02	25,776	−3.921	8.84E-05	32,937	0.595	5.52E-01
*TRHDE*	Uterus	58,537	−2.11	3.48E-02	25,600	−3.886	1.02E-04	32,937	0.612	5.40E-01
*LINC02664*	Artery tibial	58,624	2.036	4.17E-02	25,687	4.385	1.16E-05	32,937	−1.156	2.48E-01
*ADAMTSL3*	Brain cortex	58,713	2.02	4.34E-02	25,776	3.949	7.84E-05	32,937	−0.797	4.25E-01
*SH3GLB1*	Adrenal gland	58,713	1.89	5.88E-02	25,776	3.91	9.25E-05	32,937	−0.935	3.50E-01

False discovery rate adjusted genome-wide significance: *p*-value < 1.02 × 10^−4^.

**Table 2:** The genes with significant effect independent to nearby known LOAD SNPs.

			Unadjusted model			Adjusted for SNP		Adjusted for gene	
Known LOAD SNP	Genes	Tissue	*N*	*Z*	*p*-value	*Z*	*p*-value	*Z*	*p*-value
APOE (rs429358); APOE (rs7412); CD33 (rs3865444)	*LILRA5*	Lung	21,924	−5.301	1.15E-07	−4.903	9.43E-07	−5.358	8.43E-08
BIN1 (rs6733839)	*TMEM163*	Thyroid	25,776	−4.163	3.14E-05	−4.177	2.95E-05	−4.141	3.46E-05
CELF1 (rs10838725)	*C1QTNF4*	Brain cerebellar hemisphere	25,776	4.397	1.10E-05	4.055	5.01E-05	4.115	3.87E-05
	*FAM180B*	Artery coronary	25,776	−4.504	6.67E-06	−4.13	3.64E-05	−4.317	1.58E-05
	*KBTBD4*	Kidney cortex	25,776	3.996	6.45E-05	3.911	9.19E-05	3.803	0.0001431
	*MTCH2*	Skin sun-exposed lower leg	25,776	−4.245	2.19E-05	−3.969	7.23E-05	−3.783	0.000155
	*NDUFS3*	Cells cultured fibroblasts	25,776	4.261	2.03E-05	3.899	9.64E-05	4.323	1.54E-05
	*PSMC3*	Cells cultured fibroblasts	25,776	−4.263	2.01E-05	−3.89	1.00E-04	−4.3	1.71E-05
	*PTPRJ*	Brain cortex	25,776	4.356	1.32E-05	4.274	1.92E-05	3.834	0.0001259
	*RAPSN*	Stomach	25,776	4.519	6.23E-06	4.205	2.61E-05	4.39	1.13E-05
	*SLC39A13*	Adipose visceral omentum	25,776	−4.279	1.88E-05	−3.903	9.49E-05	−4.771	1.84E-06
SORL1(rs11218343)	*SIK2*	Brain cerebellar hemisphere	25,776	4.462	8.14E-06	4.44	9.00E-06	NA	NA

**Fig. 2:**
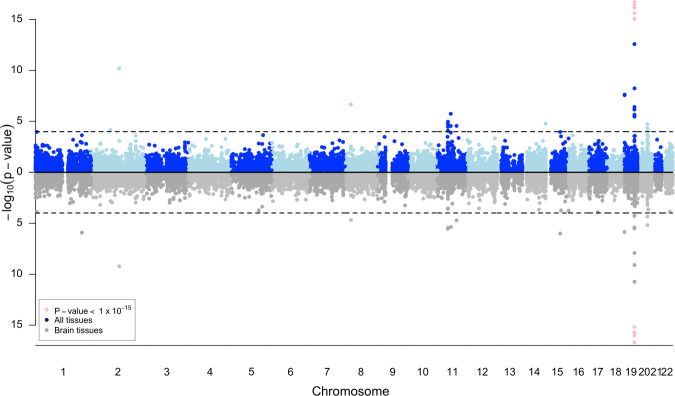
Miami plot of cross-all GTEx tissue (blue dots, top) and cross-brain tissues model (gray dots, bottom). *p*-values less than 1 × 10^−15^ (pink dots) were truncated to improve the clarity of peaks in other, less significant regions.

In addition to the tissue-specific models, we performed two cross-tissue analyses: cross-brain, including the 14 brain tissues in GTEx, and cross-all tissues, including all 49 GTEx tissues across organ systems. In the cross-all and cross-brain tissue models, 34 genes were significantly associated with LOAD; *APOE* was the most strongly associated gene in both models (*p*-value = 4.3 × 10^−^^364^ in the cross-all tissue model and *p*-value = 1.0 × 10^−^^373^ in the cross-brain model, Fig. 2 and Table 3). Notably, eight genes identified in the cross-tissue analyses were not significantly associated with tissue-specific models, including *ACOT8, ASPG, CNN2, CTSA, ABP1, ISOC2, PLTP*, and *ZSWIM1*.

**Table 3:** The significant genes in cross-tissue models.

	All GTEx tissues				GTEx brain tissues			
Genes	*N*	Tissues	PCs	*p*-value	*N*	Tissues	PCs	*p*-value
*APOE*	58,713	36.92	5.74	4.36E-364	58,713	9.72	1.59	1.00E-373
*NECTIN2*	58,713	45.18	8.23	9.22E-198	58,713	9.18	4.33	4.13E-75
*APOC1*	58,713	16.15	4.56	1.14E-165	19,246	3.00	2.00	4.02E-53
*APOC4*	58,713	9.90	3.13	1.49E-73	58,713	4.00	1.21	1.65E-88
*BCAM*	58,713	45.05	5.74	9.62E-20	57,358	12.00	2.00	8.11E-02
*APOC2*	58,713	47.28	4.95	2.90E-13	58,713	12.79	3.00	4.14E-06
*BIN1*	58,713	46.00	8.13	7.28E-11	58,713	13.00	4.92	7.03E-10
*TRAPPC6A*	58,713	26.97	3.05	7.01E-09	58,713	6.54	2.59	9.60E-10
*ABCA7*	57,358	44.32	7.68	2.94E-08	57,358	10.79	2.61	1.82E-06
*CNN2*	57,358	42.00	8.39	3.33E-08	57,358	12.34	3.79	3.91E-02
*CLU*	58,713	23.00	5.56	2.80E-07	25,776	3.00	2.00	7.16E-03
*ZNF296*	58,713	45.00	5.51	5.04E-07	58,713	10.00	2.21	2.13E-11
*CLPTM1*	58,713	31.18	3.87	8.41E-07	58,713	8.38	2.97	1.49E-08
*EXOC3L2*	58,713	29.92	4.18	2.87E-06	58,713	4.00	2.28	3.53E-03
*CEACAM19*	58,713	49.00	5.59	3.50E-06	58,713	13.00	1.05	4.97E-06
*SLC39A13*	58,713	47.00	3.77	1.45E-05	58,713	12.00	2.00	3.57E-04
*ASPG*	58,713	41.33	7.00	2.22E-05	58,713	11.51	3.64	8.72E-02
*CTSA*	58,713	19.10	4.67	2.50E-05	58,713	4.44	2.00	1.37E-02
*ACP2*	58,713	49.00	3.18	2.59E-05	58,713	13.00	2.03	3.86E-06
*PICALM*	58,713	41.00	5.00	3.72E-05	58,713	8.00	4.00	2.56E-05
*PSMC3*	58,713	44.00	3.05	4.54E-05	58,713	11.00	2.00	4.62E-06
*ZSWIM1*	58,713	32.00	4.49	6.49E-05	58,713	9.00	2.00	4.71E-03
*FABP1*	58,713	43.00	2.85	9.56E-05	58,713	13.00	2.62	7.91E-02
*ACOT8*	58,713	47.74	3.18	1.05E-04	58,713	12.90	2.26	6.21E-05
*APH1B*	58,713	47.59	4.41	1.49E-04	58,713	11.79	3.03	1.28E-06
*MS4A4E*	58,713	29.97	2.87	1.69E-04	25,776	3.00	1.00	5.62E-06
*CR1*	58,713	25.03	3.03	3.16E-04	58,713	9.21	1.41	1.55E-06
*PVR*	58,713	48.00	7.54	3.77E-04	58,713	12.00	2.92	7.14E-05
*PLTP*	58,713	44.41	6.23	1.38E-03	58,713	13.00	4.28	8.58E-06
*SCARA3*	58,713	36.21	8.00	3.97E-03	58,713	9.00	4.64	2.73E-05
*DMWD*	58,713	39.95	5.23	5.22E-03	58,713	6.00	2.00	1.11E-02
*SYMPK*	58,713	38.72	6.18	1.17E-01	58,713	7.00	2.03	8.97E-03
*EML2*	58,713	30.56	9.15	1.87E-01	57,358	7.21	3.71	5.91E-02
*ISOC2*	57,358	38.21	3.11	9.12E-01	56,328	7.84	2.38	8.84E-01

To assess causal inference, we used the GReX fine-mapping method FOCUS to identify the most likely causal genes, testing 34 genome regions across 49 tissues. The PIP was used to evaluate the potential of our observed GReX association representing a causal effect, and it was strongly correlated with the *Z*-score from our GReX association tests (rho = 0.758). Among the tested GReX signals, 152 from 21 unique genes were considered likely causal (PIP > 0.9, Supplementary Table 5). We also used Mendelian randomization to examine if the association from eQTL to LOAD is mediated by gene expression. Among 1395 identified LOAD-related GReX, 556 signals from 107 unique genes reached statistical significance (Benjamini & Hochberg FDR-adjusted *p*-value < 0.05, Supplementary Table 6), indicating that the effect of these eQTLs on disease status is through their impact on gene expression.

**Fig. 3:**
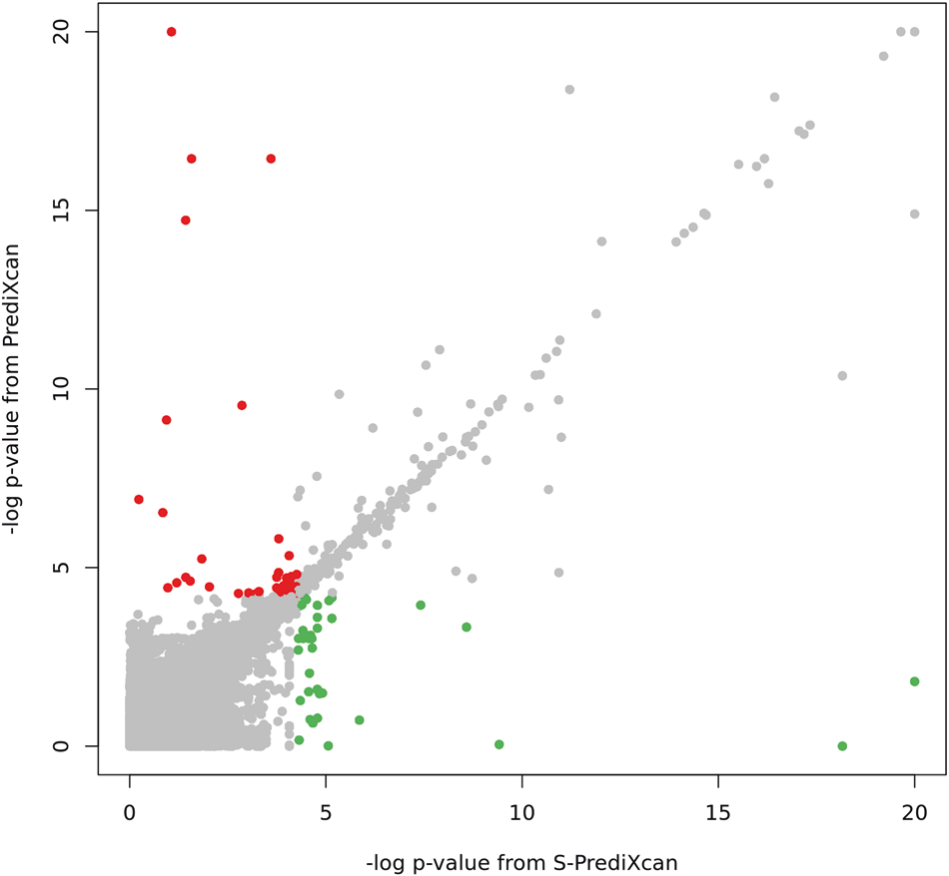
Scatter plot of log-transformed *p*-values from SNP-based (PrediXcan) and summary statistic (S-PrediXcan) GReX approach. Each dot shows the gene-level *p*-value from a SNP-based approach (PrediXcan, y-axis) and summary statistic approach (S-PrediXcan, x-axis). Color indicates that the signal (tissue-specific GReX) was only identified in PrediXcan (red, FDR < 0.05) or only in S-PrediXcan (green, FDR < 0.05).

**Fig. 4:**
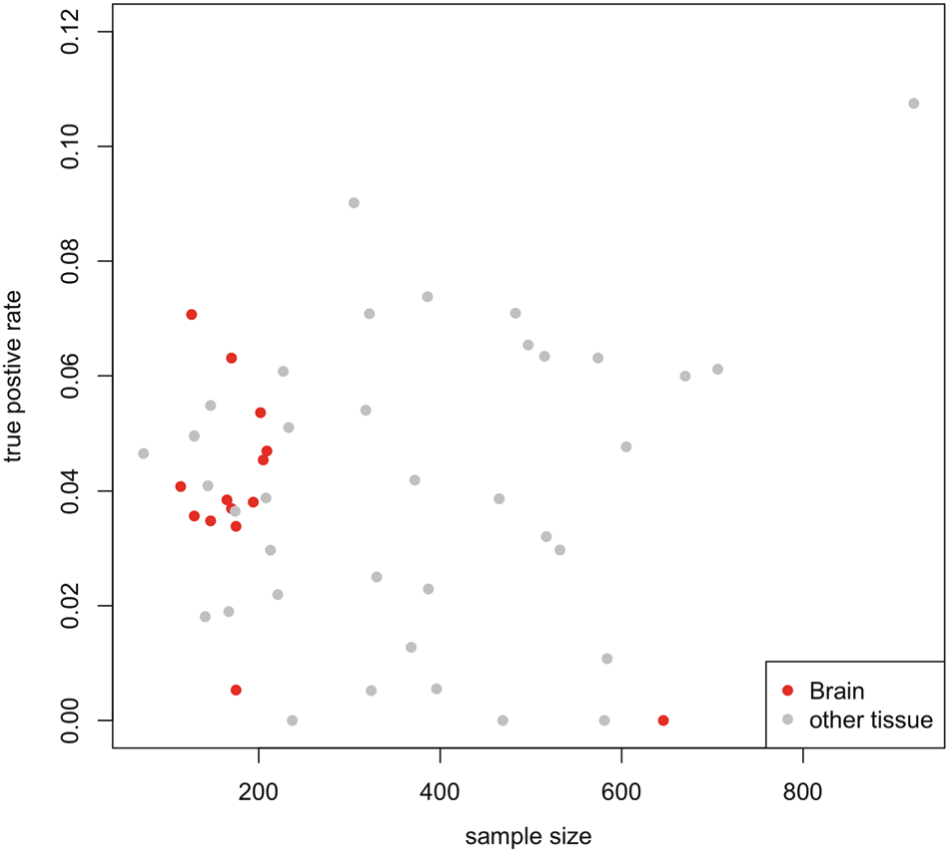
Scatter plot of sample size used for model building against the true positive rate. X-axis represents the number of subjects used in PrediXcan model building and Y-axis represents the true positive rate for each model in our analysis. Color represents tissue source (red for brain tissues and gray for other tissues).

Furthermore, we compared the sensitivity between PrediXcan and S-PrediXcan in ADGC (Fig. 3). Overall, we observed a high correlation between their log-transformed *p*-values (*r*^2^ = 0.90). However, 66 signals were significant (FDR-adjusted *p* < 0.05) only in PrediXcan, and 47 were only identified in S-PrediXcan, highlighting the subtle change in power due to slight differences between the in-sample and reference sample linkage disequilibrium patterns (Supplementary Fig. 1). We also used the true positive rate to evaluate the sensitivity of each tissue-specific model (Fig. 4). The brain tissues had a similar true positive rate (π1, see “Subjects and methods” section) compared to other tissues (mean = 0.039 in 14 brain tissues and 0.041 in others, *p*-value = 0.736), and the true positive rate was not significantly correlated with the sample size for model building (*r*^2^ = 0.144, *p*-value = 0.311).

We calculated LOAD heritability using summary statistics from a previous LOAD GWAS [16]. Overall genome-wide heritability from this study was 6.8%. We then estimated several partitions of heritability based on our GReX findings. This resulted in three primary findings. First, the 216 identified LOAD genes contain only 7.1% of the SNPs used for heritability estimation, but explain 60.9% of heritability, representing a significant enrichment (*p*-value = 1.3E–04, Table 4). Among these genes, implicated genes in the *APOE* region (comprising 0.5% of SNPs) explain 16.1% of heritability, and the genes outside of this region (comprising 6.6% of SNPs) explain 44.8%. Second, we estimated the heritability from the brain and blood tissues alone. The brain tissue models identified more LOAD genes (117 genes) than blood tissues (62 genes) and explained slightly higher heritability (47.4% in the brain vs. 43.1% in the blood). Third, we estimated heritability using the 22 LOAD SNPs which were previously used to generate an optimal polygenic risk score model for LOAD [60] and SNPs in the surrounding ± 5 Kb regions; these SNPs explained 16.8% of heritability.

**Table 4:** Partitioned of the SNP-based heritability of LOAD.

Category	SNPs (%)	*h*^2^ (%)	SE	Enrichment^a^	SE	*p*-value
ALL SNPs	100.0	100.0	0.1%	1		
AD genes	7.1	60.9	11.4%	8.62	1.61	1.3E–04
APOE region	0.5	16.1	12.7%	32.02	25.18	0.2474
Non-APOE region	6.6	44.8	6.5%	6.83	0.99	1.9E–19
Brain tissue	4.5	47.4	10.1%	10.63	2.27	3.9E–04
Blood tissue	2.7	43.1	12.1%	15.91	4.46	4.7E–03
Previous GWAS	0.008	16.8	7.4%	2039.62	900.05	0.0189

The “All SNPs” category used all available SNPs to estimate the heritability of LOAD, and defines 100% of the SNP-based heritability; AD genes used all SNPs located within ±1 Mb of our 216 identified AD genes; previous GWAS used 22 SNPs, which were used to generate an optimal polygenic risk score model [60], and their surrounding SNPs within ±5 Kb.

^a^Enrichment = Proportion of heritability/proportion of SNPs

## Discussion

To further understand the genetic mechanisms of LOAD, we performed functionally oriented association analyses targeting tissue-specific and cross-tissue effects on LOAD-risk. We applied GReX models in a total of 51 tissues to 20,613 LOAD cases and 37,658 controls. As a result, 72 novel genes and 136 genes from known LOAD regions were identified after study-wide multiple test correction (FDR < 0.05). In addition, we applied both cross-brain-tissues and cross-GTEx models and we found eight additional genes significantly associated with LOAD. Secondly, we compared the SNP-based GReX approach to the summary statistic-based approach, and demonstrated the benefit of SNP-based methods, which leverage the internal LD structure of the empirical data instead of an external panel. Finally, we estimated the proportion of LOAD SNP-based heritability that can be captured by our identified LOAD genes to represent the improvement of GReX analysis over traditional GWAS. Our results suggest over 60% of LOAD heritability can be captured by our 216 identified LOAD genes.

Most previously described LOAD genes have been identified through GWAS, and we observe that for eight known genes, *APOE, BIN1, CLU, PTK2B, CR1, MS4A2, PICALM, IQCK*, and *TREML2*, their genetically regulated expression levels were significantly associated with LOAD status as well. Several genes close to these known genes were also significantly associated with LOAD. For instance, results for *TOMM40* in GTEx skin tissues were the strongest association signals in all analyses; this strong effect observed in skin tissues may be due to shared genetic regulation between skin and brain tissues and increased power due to more available expression data in skin tissues. Notably, skin biopsies have been suggested as an approach to detect dysregulated or abnormal protein levels in Alzheimer’s disease between healthy participants, participants with LOAD, and participants with dementia due to other causes [61]. *TOMM40* encodes Tom40 protein, Translocase of the Outer Mitochondrial Membrane, 40 kD, and is located in a region in high linkage disequilibrium with *APOE*. Association of *TOMM40* variants with LOAD has been observed in previous genetic studies [62], and several studies indicate that the effect of *TOMM40* is independent of *APOE* [63–65]. Lower expression of *TOMM40* in blood has also been reported in LOAD cases [66], with a consistent direction of effect with our results in the DGN whole blood model. Furthermore, we adjusted for the known LOAD variants representing the *APOE* ε2/ε3/ε4 haplotype reported in Naj et al. [41] in conditional analysis of this locus, and results indicate that the effect of the genetically regulated expression on LOAD was driven mostly by the index variants at *APOE* (rs7412, rs429358, rs145999145, and rs3865444). We used FOCUS to clarify the causal inference of identified genes from known LOAD regions, and *TOMM40* was also identified as a likely causal gene in many tissues, e.g., muscle, brain cortex and pituitary gland. In addition, our Mendelian randomization results also suggest that the effect of eQTLs of *APOE* on LOAD were not significantly mediated by the expression of *APOE* in many tissues, including both skin and muscle tissues. These results suggest that previously identified *APOE* SNPs may impact the risk of LOAD through coregulation of the expression of nearby genes, such as *TOMM40*. We observed a similar pattern throughout our results, indicating that previously identified LOAD SNPs may co-regulate the expression of several nearby genes rather than only the nearest gene. After adjusting the nearby known LOAD SNPs, we still can identify 11 unique genes that retained significance, including *PTPRJ* from the *CELF1* region. *PTPRJ* encodes a protein of the family of tyrosine phosphatase, and it has been reported as a shared genetic factor of LOAD and major depression disorder [67].

However, we also identified 72 novel independent genes that are >10 MB from previously reported signals, including *TRIT1*, *TSPAN14*, and *MTDH*. *TRIT1* encodes TRNA Isopentenyltransferase 1, which is targeted to mitochondria and modified the transfers RNA. *TRIT1* has been known as a tumor suppressor for several forms of cancer [68], and its pleiotropy between LOAD and major depressive disorder [67] or cardiovascular risk factor has been previously noted [69]. *TSPAN14* is a member of tetraspanins, a family of compact and glycosylated transmembrane proteins. Previous research has reported tetraspanins’ function on the precursor of amyloid-beta (Aβ) peptide, amyloid precursor protein, and their potential roles in the development of LOAD [70]. In addition, *MTDH* encodes metadherin, which is also known as astrocyte elevated gene-1 protein (AEG-1). The role of AEG-1 in tumor progression and neurodegeneration, especially HIV-induced dementia, has been previously reported [71, 72]. The GReX-based analysis has been applied in several previous small LOAD studies [20, 73, 74]. With our larger sample size and updated models, we successfully replicated their findings of known LOAD genes, and identified additional novel genes (Supplementary Table 4).

To explore which tissues contribute the most significant effects to LOAD-risk, we also evaluated the true positive rate for each tissue-specific model using the methods outlined in Storey et al. [57]. Our findings suggest that non-brain tissues, particularly whole blood, are important for detecting GReX effects on LOAD and may imply the relevance of non-brain tissue in LOAD pathology. Given the difficulty of characterizing transcriptomic effects in brain tissue, the utility of alternate, accessible tissues in understanding LOAD pathogenesis will be key to future studies.

Motivated by the extensive observed shared genetic regulation of gene expression across tissues [37], we conducted cross-tissue analyses in addition to tissue-specific analyses. If a gene is tissue-specific in its regulation, then a tissue-specific model rather than a cross-tissue model, should have a better power to detect its effect. However, if patterns of expression suggest that a gene has shared regulation across many tissues and effects are small but consistent across many tissues that may have small sample sizes in GTeX (e.g., brain tissues), then a cross-tissue model would be expected to have greater power than a single-tissue model. A total of 34 genes were identified in our cross-tissue analyses, eight of which were only identified in cross-tissue analyses, while 188 were only identified by single-tissue analysis. These results indicate that cross-tissue analysis can detect additional significant associations at genes where the effects are consistent across tissues, because cross-tissue and tissue-specific analyses may provide complementary information.

Moreover, we compared the use of SNP-based GReX analysis, PrediXcan, and summary statistic-based GReX analysis, S-PrediXcan, in the same dataset and found that the SNP-based approach identified more significant signals. Since summary statistic-based GReX prediction approaches rely on an external linkage disequilibrium panel, the reduced significance of the summary statistic-based method may be caused by differences in linkage disequilibrium between the external panel and the population in which the summary statistics were derived. Though these differences in linkage disequilibrium are likely subtle, our findings suggest that although the shifts in *p*-values were not large, for borderline signals, S-PrediXcan may have slightly reduced power to detect true effects. We note, however, that careful simulation will be needed to fully characterize the impact of differences in linkage disequilibrium on the power of these approaches, which is beyond the scope of the present study.

To further validate our findings, we estimated the proportion of LOAD heritability that can be explained by the identified genes. Our results indicate that our 216 LOAD genes explain a significantly enriched proportion of heritability (enrichment = 8.62, *p*-value = 1.3 × 10^−4^, Table 4). For comparison, we also calculated heritability explained by the 22 LOAD SNP loci (±5 kb) included in a recent LOAD polygenic risk score model [60]. Although the loci containing the 22 SNPs used in LOAD PGRS are strongly enriched, our identified LOAD gene set explains more than three times the SNP-based heritability of LOAD, demonstrating the benefit of applying the functionally oriented approach to investigate the pathology of LOAD. Furthermore, we compared the proportion of explained heritability by the identified genes from brain tissue and blood tissue. Even though the brain is considered to be the causal tissue for LOAD, the blood tissues can capture similar heritability with brain tissues, and it may emphasize the importance of non-brain tissue in LOAD again. Also, a previous study demonstrated that the blood transcriptome can well predict the genes’ expression in other tissues [75], and it may offer another explanation about our highly explained LOAD heritability from blood.

There are several limitations of the present study. The gene expression prediction models are primarily based on GTEx data, and our power to detect associations is limited somewhat by tissue-specific sample sizes in GTEx. In particular, limited sample sizes for the brain tissues in GTEx reduced our ability to identify associations between LOAD-risk and genes with tissue-specific expression in the brain. To mitigate this limitation, we included models built in the CommonMind data. Also, some genes have expression patterns dependent on age [76], and so our predicted genetically regulated expression profile may reflect the age of the GTEx reference samples instead of the advanced ages associated with LOAD, limiting our power to detect such effects. Furthermore, our analysis examines the association of predicted genetically regulated expression, rather than directly measured expression levels. These imputed levels may differ from true, measured expression, which is affected by other environmental or temporal factors [77]. Although we may not perfectly impute the true gene expression levels or capture the effect of gene-environment, the models of genetically regulated expression that we applied here offer an opportunity to investigate age- and environment-independent effects of gene regulation on LOAD. Finally, our study populations are entirely European ancestry, limiting our ability to detect some loci with effects that vary by population and the generalizability of our results to other populations. Further study in individuals of non-European ancestry is needed to ensure that this limitation does not persist and contributes to health disparities.

In conclusion, analyzing genetic data from 20,613 LOAD cases and 37,658 controls from 39 epidemiological studies of LOAD, we profiled the genetically regulated expression genome-wide in 51 diverse tissues and expression data from GTEx, CommonMind, and DGN using SNP-based and summary statistics-based GReX approaches. Many of our significant gene-based associations fall within 10 Mb distance of previously described loci, suggesting co-regulation by GWAS-identified SNPs. In addition, we identify 72 novel genes from either tissue-specific analysis or cross-tissue analysis. Together, the 216 LOAD genes we identify explain over 60% of the SNP-based heritability of LOAD in these data. While the role of genes with differentially regulated expression levels in LOAD progression is still unclear, and more functionally oriented genetic studies of LOAD are needed, these findings highlight the power of expression prediction approaches to identify novel genes.

## Supplementary information

supplementary tables and figure

Supplementary information

## Data Availability

The data sets used and/or analyzed during the current study are available from the corresponding author on reasonable request.

## Code availability

All code is available on GitHub https://github.com/belowlab/LOAD-GReX/.
